# Ectopic Recruitment of the CTCF N-Terminal Domain with Two Proximal Zinc-Finger Domains as a Tool for 3D Genome Engineering

**DOI:** 10.3390/ijms26157446

**Published:** 2025-08-01

**Authors:** Eugenia A. Tiukacheva, Artem V. Luzhin, Natalia Kruglova, Anastasia S. Shtompel, Grigorii Antonov, Anna Tvorogova, Yegor Vassetzky, Sergey V. Ulianov, Sergey V. Razin

**Affiliations:** 1 Institute of Gene Biology, Moscow 119334, Russia; 2 Koltzov Institute of Developmental Biology, Moscow 119334, Russia; 3 Department of Biological and Medical Physics, Moscow Institute of Physics and Technology, Moscow 141700, Russia; 4 CNRS UMR9018, Institut Gustave Roussy, 94805 Villejuif, France; 5 Department of Molecular Biology, Faculty of Biology, Lomonosov Moscow State University, Moscow 119991, Russia; 6 Center for Precision Genome Editing and Genetic Technologies for Biomedicine, Institute of Gene Biology, Russian Academy of Sciences, Moscow 119334, Russia

**Keywords:** CTCF, cohesin, loop extrusion, chromatin loops, genome spatial organization, 3D genome engineering

## Abstract

Enhancer-promoter interactions occur in the chromatin loci delineated by the CCCTC-binding zinc-finger protein CTCF. CTCF binding is frequently perturbed in genetic disorders and cancer, allowing for misregulation of genes. Here, we developed a panel of chimeric proteins consisting of either full-length or truncated CTCF fused with programmable DNA-binding module dCas9 and fluorescent tracker EGFP. We found that the recruitment of a chimeric protein based on the CTCF N-terminal domain and two zinc-finger domains to the human *HOXD* locus leads to the de novo formation of a spatial contact with a nearby cohesin/CTCF-bound region, anchoring several chromatin loops. This chimeric protein did not show binding to CTCF motifs and did not affect the epigenetic and transcription profile of the locus. Recruitment of this chimeric protein is also able to restore chromatin loops, lost after deletion of an endogenous CTCF-binding site. Together, our data indicate that the ectopic recruitment of the CTCF N-terminal part could be an appropriate tool for 3D genome engineering.

## 1. Introduction

The interphase mammalian genome is hierarchically organized into spatial structures [1] varying in size, mechanisms of formation, and degree of variability between individual cells in a population [2,3] and between different taxa [4]. The question of the relationships between these structures and the regulation of gene expression is particularly important in light of the expanding list of diseases caused by changes in gene expression at loci with violations of genome topology [5]. Studying the possibility of preventing the effects of such alterations opens up broad prospects in the correction of pathological phenotypes driven by developmental abnormalities and–potentially–in the treatment of at least some cancers [6].

Compartmentalization driven by homotypic association of active and repressed chromatin [7], and loop extrusion mediated by structural maintenance of chromosomes (SMC) protein complexes such as cohesin and condensin [8] are the major determinants of the 3D genome in mammals [9]. Compartmentalization reflects large-scale spatial segregation of active and repressed genome regions within the nuclear space. In contrast, extrusion, acting exclusively in cis, links genes and regulatory elements located at a distance of up to several megabases from each other. Most of the found examples of pathologies associated with changes in the genome topology are violations of the boundaries of topologically associated domains (TADs) and contact loops, which are thought to be predominantly formed by loop extrusion [10].

Extrusion is an ATP-dependent translocation of the cohesin complex along DNA accompanied by a progressive growth of a DNA loop [11,12]. The movement of extrusion complexes is limited by various barriers. A zinc-finger DNA-binding protein, CTCF, plays the major role in cohesin stalling [13,14,15], although other barriers such as condensates, MCM complexes [16] and transcription complexes also exist [17]. The human genome contains approximately 22,000,000 CTCF-binding motifs [18]; ~1% of them are bound by CTCF in different cell types [18] (hereinafter, they are referred to as CTCF-binding sites, CBSs). CTCF motifs are not palindromes, so the direction of the motif (“forward” or “reverse”) dictates the CTCF positioning on DNA: N-terminal and C-terminal domains of the protein are located at 3′- and 5′-termini of the motif, respectively. Extrusion termination typically occurs when the extruding cohesin collides with a CBS where CTCF is bound to DNA in such a way that its N-terminal domain faces the moving cohesin. Accordingly, the majority of CTCF/cohesin-mediated loops are anchored by convergently orientated CTCF motifs [19]. CBSs are frequently located in the vicinity of regulatory elements; thus, extrusion termination at these CBSs mediates enhancer-promoter contacts [20,21,22].

CTCF consists of unstructured N- and C-terminal domains, and 11 C2H2-zinc-finger (ZF) domains, different in amino acid composition and functions, including a module of ZF3-ZF7 responsible for the binding to a core DNA motif [23,24], and ZFs 9–11 which bind additional DNA sequence located near the core motif. Structural studies have shown the importance of the CTCF N-terminal domain for the cohesin binding and chromatin looping [13,25,26]. The YDF motif (226–228 aa) is necessary for the interaction with an evolutionarily conserved structural motif located at the surface formed by STAG1 and RAD21 cohesin subunits [13,26]. Interestingly, the YDF motif (or similar FGF motif) is also found in some other chromatin-associated cohesin-interacting proteins (MCM3, WAPL) [16,26]. Linker between the N-terminal domain and the first ZF (261–264 aa) is also important for loop formation [13]. RNA-binding regions of CTCF, located at the first and tenth ZFs, affect chromatin looping at least within some genome loci [27]. Also, CTCF contains several poly(ADP-ribosyl)ation sites, located near the YDF motif [28]. This post-translational modification (PTM) is necessary for CTCF-DNA interaction, and, consequently, impacts the extrusion process [29,30]. Several other PTMs were also found in CTCF: SUMOylation in N- and C-terminal domains (K74 and K698, respectively), phosphorylation (T374 and S402 in linkers between ZF4/5 and ZF5/6, respectively, S224 in N-terminal domain, S612 in C-terminal domain) and acetylation at K20 of N-terminal domain [31,32,33,34,35]. However, their role in the cohesin stalling remains mostly unexplored. Despite extensive studies, the CTCF fragment, which is “necessary and sufficient” for the extrusion termination and loop formation, has not been identified to date.

CBS mutations, methylation, deletions, insertions and inversions have been applied to modify the spatial structure of several well-characterized genome loci [36,37,38,39,40,41,42,43]. While CBS integrity, location and orientation directly control loop formation, genetic manipulations are often challenging because they require clonal selection and are potentially fraught with side effects caused by off-target DNA breaks [44]. Thus, “non-invasive” CTCF recruitment to a locus of interest without affecting the DNA sequence is an attractive task in 3D genome engineering. To our knowledge, three strategies for the ectopic CTCF recruitment have been published. Two of them utilize a full-length CTCF, which is directed to a target site being fused with enzymatically deficient Cas9 (dCas9) [20] or via the SunTag system [21]. In the third study, a truncated form of CTCF was fused to artificial DNA-binding ZF-domains [25].

Here, we developed five chimeric proteins consisting of the full-length human CTCF protein and its subfragments fused with dCas9, and tested their ability to effectively modify the spatial structure of the *HOXD* locus in K562 cells. This cell line is extensively studied in terms of chromatin epigenetic state and spatial organization, and the chromatin loop profile of the *HOXD* locus is well-documented in different cell types and biological conditions. This provides a basis for the rational design of the experiments with the recruitment of chimeric proteins. We found that the usage of a full-length CTCF results in a pronounced off-target binding to endogenous CBSs throughout the locus. Moreover, this protein is characterized by remarkable cytotoxicity. In contrast, the chimeric protein composed of the CTCF N-terminal domain and the first two ZFs did not exhibit off-target binding and was efficiently recruited to the target site, which resulted in the formation of a chromatin loop with one of the nearby endogenous CBSs. Further, we demonstrated that this protein could be used to restore a normal loop profile that has been perturbed by the deletion of an endogenous CBS. Taken together, our data suggest that dCas9-mediated recruitment of the CTCF N-terminal part could be an effective and precise tool for 3D genome engineering.

## 2. Results

### 2.1. Construction and Expression of the CTCF-Based Chimeric Proteins

**Figure 1:**
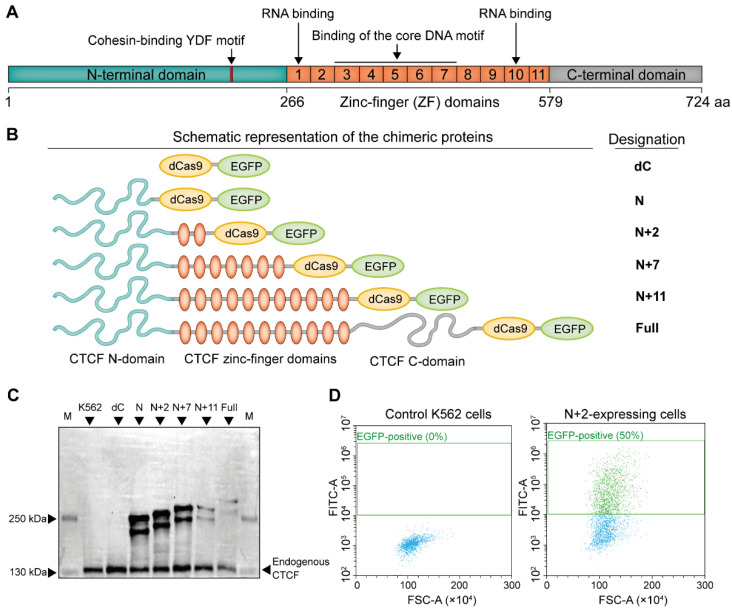
Design and expression of CTCF-dCas9-EGFP chimeric proteins. (**A**) Schematic representation of the human CTCF domain organization. Location of the N-terminal, C-terminal and zinc-finger (ZF) domains relative to the amino acid (aa) sequence is shown (according to [13,25,49]). Structural features important for chromatin looping are indicated. (**B**) Schematic representation of the chimeric proteins developed in this work. (**C**) Western Blot analysis of the chimeric protein expression in K562 cells electroporated with the corresponding expression plasmids. Antibodies against the CTCF N-terminal domain were used. Calculated molecular weights of chimeric proteins N, N+2, N+7, N+11 and Full are 209, 223, 240, 254 and 270 kDa, respectively. The presence of the second band for all chimeric proteins is potentially attributed to the poly(ADP-ribosyl)ated form [28,50]. Endogenous CTCF migrates as a 130-kDa protein and serves as a loading control. K562—control non-electroporated cells. (**D**) Flow cytometry analysis of the control and N+2-expressing K562 cells showing the proportion of EGFP-positive (FITC-A) and live (FSC-A) cells.

We constructed a panel of expression vectors encoding chimeric proteins, consisting of human CTCF (Figure 1A) fused to dCas9 and EGFP under control of MSCV LTR promoter (Supplementary Figure S1A). Previous observations suggest that the N-terminal domain of CTCF is crucial for the cohesin trapping [13,25,26,45,46], and ZFs could also be important for the chromatin looping and RNA binding [25,27,47]. Poly(ADP-ribosyl)ation sites and linker between N-terminal domain and first ZF seem to be important for interaction with cohesin [13,27]. Taking this into account, we fused dCas9-EGFP with either full-length CTCF CDS or its several truncated forms composed of a part of the N-terminal domain lacking major Poly(ADP-ribosyl)ation sites, and the entire N-terminal domain together with 2, 7 or 11 ZF-domains (hereinafter are referred to as Full, N, N+2, N+7 and N+11, respectively) (Figure 1B). Western Blot analysis with antibodies raised against the CTCF N-terminal domain confirmed the expression of all chimeric proteins (Figure 1C). We noticed that the expression level of N, N+2 and N+7 variants is comparable (or even higher) to that of endogenous CTCF. However, N+11 and Full chimeric proteins were significantly less abundant (Figure 1D, Supplementary Figure S1B) as the viability of N+11- and Full-expressing cells was remarkably reduced, as compared to other constructs (Supplementary Figure S1B,C). These results agree with the previous report [48].

### 2.2. N+2 Chimeric Protein Effectively and Specifically Binds to the Target Site

**Figure 2:**
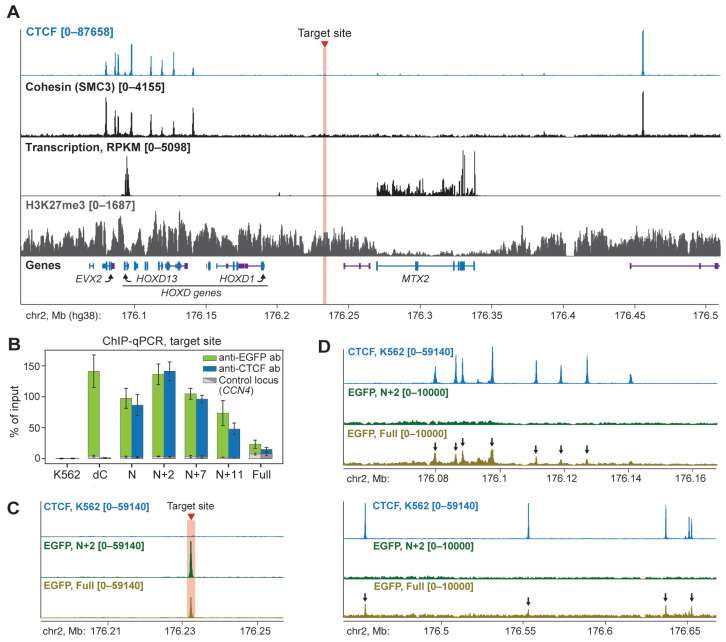
N+2 chimeric protein effectively and specifically binds to the target site. (**A**) A snapshot from the UCSC Genome Browser [54] showing profiles of CTCF, cohesin, H3K27me3 and transcription across the *HOXD* gene locus (RPKM). Note that in K562 cells, *HOXD* genes are typically Polycomb-repressed and extensively marked with H3K27me3. However, in the used clone of K562 cells, *HOXD13* expression level is relatively high, comparable to that of the housekeeping *MTX2* gene. Protein-coding and non-coding RNA genes are highlighted in blue and violet, respectively. (**B**) ChIP-qPCR analysis of the chimeric protein binding at the target site. An amplicon within the *CCN4* locus was used as a negative control. The average value of two independent biological replicates and the standard error of the mean are shown. (**C**) ChIP-seq analysis (RPKM) of N+2 and Full chimeric protein binding at the target site. Upper line: control K562 cells, anti-CTCF antibodies were used; middle and bottom lines: N+2- and Full-expressing cells, anti-EGFP antibodies were used. (**D**) Representative examples of the off-target binding of the Full chimeric protein at the endogenous CBSs (black arrows) within the *HOXD* locus revealed by ChIP-seq (RPKM). Upper lines: control K562 cells, anti-CTCF antibodies; middle and bottom lines: N+2- and Full-expressing cells, anti-EGFP antibodies.

Before testing the ability of chimeric proteins to establish chromatin loops de novo, we performed an epigenetic and transcription profiling of the *HOXD* locus and surrounding chromosome regions in K562 cells. We performed chromatin immunoprecipitation (ChIP-seq) with anti-CTCF, anti-RAD21 and anti-H3K27me3 antibodies and RNA-seq. Libraries were enriched with fragments from the 175,634,800–176,937,632 bp region of chr2 via hybridization with baits prepared from bacterial artificial chromosomes covering this region [51] (Supplementary Table S1; see Methods for the details). Consistent with publicly available data, we have observed extensive binding of CTCF and cohesin at the *HOXD* locus (Figure 2A). We also confirmed that the locus and its vicinity were marked with a Polycomb-mediated H3K27me3 chromatin mark. Surprisingly, we detected a high level of *HOXD13* gene transcription, which is normally inactive in K562 cells. This feature of our K562 batch illustrates previously reported epigenetic and transcriptional heterogeneity of different clones of commonly used laboratory cell lines [52,53].

Based on these profiles, we selected chr2:176,232,117–176,232,185 bp (hg38) genomic positions for the targeted recruitment of the chimeric proteins (Figure 2A, hereinafter this location is referred to as the target site, marked with a red line). This region is located far away from the nearest CTCF/cohesin-binding sites (>80 kb) within the H3K27me3-enriched intergenic area. This allowed us to reliably analyze chromatin looping between the target site and endogenous CBSs (see below).

We co-transfected expression vectors for each chimeric protein with the plasmids encoding two gRNAs complementary to both DNA strands (Supplementary Table S2). ChIP-qPCR analysis with antibodies raised against EGFP and CTCF N-terminal domain has revealed binding of all chimeric proteins to the target site (Figure 2B, Supplementary Table S3). We should note that the difference in the ChIP signal value between chimeric proteins reflects the percentage of expressing cells (Supplementary Figure S1A) rather than the different effectiveness of the recruitment. Next, to check the off-target binding, we performed ChIP-seq analysis with anti-EGFP antibodies for the N+2- and Full-expressing cells and observed a single strong peak exactly at the target site in the case of N+2 chimera (Figure 2C). However, the Full chimeric protein was also bound to endogenous CBSs throughout the locus and potentially, genome-wide (Figure 2D, Supplementary Figure S2). This demonstrates that full-length CTCF retains its natural DNA-binding activity when fused to dCas9 and EGFP, which likely interferes with the recruitment to the target site and could increase the CTCF abundance at genomic CBSs.

### 2.3. N+2 Chimeric Protein Mediates Cohesin-Dependent Chromatin Looping

**Figure 3:**
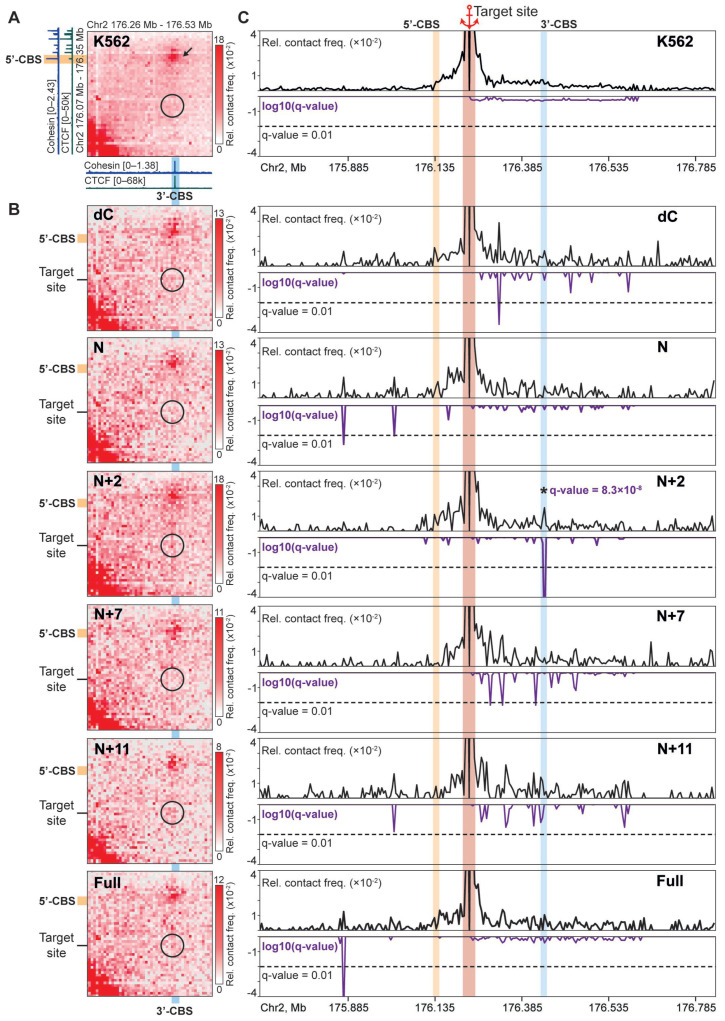
N+2 chimeric protein mediates chromatin looping. (**A**) A snapshot of the C-TALE heatmap (5-kb resolution) for the control K562 cells, showing a loop between 5′-CBS and 3′-CBS (black arrow). A black circle designates an area of the map where a contact between the target site and 3′-CBS is expected. The color intensity is directly proportional to the C-TALE-captured contact frequency (see the color bars to the right of the heatmaps). (**B**) The same fragments of the C-TALE heatmaps were obtained from the K562 cells expressing dC, N, N+2, N+7, N+11 and Full chimeric proteins. (**C**) Virtual 4C profiles of the relative contact frequency of the target site (top panels) and FDR-adjusted *p*-values (q-values) of the contacts (violet plots in the bottom panels). The lower the q-value, the higher the statistical significance of the difference between the observed and expected contact frequency. The significance threshold equal to 0.01 is indicated by a dashed line. A contact between the target site and 3′-CBS in N+2-expressing cells is indicated with an asterisk.

Next, we applied the C-TALE technique [51] to analyze chromatin contact patterns within the *HOXD* locus upon recruitment of the chimeric proteins. We frequently used the frequently cutting DpnII restriction enzyme to digest genomic DNA and sequenced libraries with 12.2–30.9 million paired reads to obtain 101,000–870,000 unique contacts (Supplementary Table S4) and to construct maps at 5-kb resolution (Figure 3A,B, Supplementary Figure S3).

It should be noted that, in these experiments, we have anticipated the formation of chimeric protein-mediated contacts between the target site and nearby CTCF/cohesin-occupied regions (5′- and 3′-CBS, Figure 3A), which are endogenous barriers for the extrusion. As expected, we did not observe loops between the target site and any other region of the locus in control K562 cells (Figure 3A). We also found that the recruitment of dCas9 alone (dC) did not result in the appearance of a contact between the target site and nearby CBSs (Figure 3B,C). This is in agreement with the previous observation suggesting that dCas9 could possess only a weak (if any) boundary activity for the cohesin-dependent extrusion in vivo [20,55], while effectively stalls cohesin in vitro [13]. Preliminary visual inspection of the maps revealed that, among chimeric proteins, only N+2, N+11, and Full variants promoted a moderately increased contact frequency between the target site and 3′-CBS (Figure 3B). To assess this effect quantitatively and to avoid misinterpretation of the relatively noisy C-TALE heatmaps, we have constructed virtual 4C profiles of the target site and calculated the FDR-adjusted p-value (q-value) as a measure of statistical significance of the observed contacts (see Methods for the details, Figure 3C).

**Figure 4:**
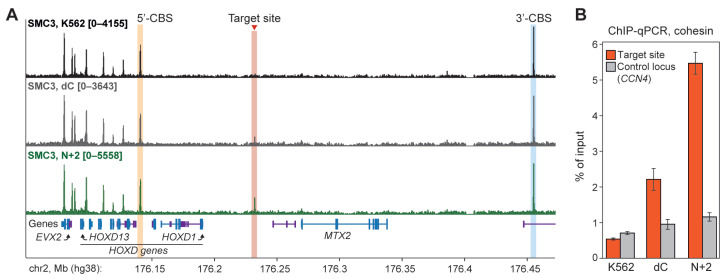
The recruitment of N+2 chimeric protein leads to the accumulation of cohesin at the target site. (**A**) SMC3 ChIP-seq profiles (RPKM) obtained from control K562 cells (upper line) and from cells expressing dC (middle line) and N+2 chimeric proteins (bottom line). Protein-coding and non-coding RNA genes are highlighted in blue and violet, respectively. (**B**) Cohesin accumulation level at the target site as revealed by ChIP-qPCR with anti-SMC3 and anti-RAD21 antibodies (used in different biological replicates). The average value of two independent biological replicates and the standard error of the mean are shown.

This analysis revealed that the recruitment of N+2 chimeric protein, in contrast to other variants, including the Full one, results in the formation of a highly statistically significant (q = 8.3 × 10^−8^) contact between the target site and 3′-CBS. This was confirmed in a reciprocal analysis where the 4C-viewpoint has been placed at the 3′-CBS (Supplementary Figure S4). Strikingly, ChIP-seq profiling showed that the N+2 binding results in cohesin accumulation selectively at the target site to a level comparable to some CBSs within the *HOXD* cluster (Figure 4A). This was confirmed by ChIP-qPCR with an amplicon placed at the target site (Figure 4B). This potentially indicates that the N+2 protein possesses a barrier activity for the cohesin-driven extrusion. A remarkable feature of the target site interaction profile in the N+2-expressing cells is the absence of a contact with 5′-CBS (Figure 3C). Together, these data indicate that contacts between the target site and nearby CBSs are established by the extrusion starting within a linker between the target site and 3′-CBS, but not 5′-CBS.

We have also found that the N+2-mediated looping between the target site and 3′-CBS did not affect the H3K27me3 level across the entire locus (Supplementary Figure S5A), with a moderate decrease in the immediate vicinity of the target site. The last could be explained by the observation that dCas9 binding potentially destabilizes nucleosomes at least on some DNA sequences [56]. Correspondingly, RNA-seq analysis did not detect any statistically significant changes in gene expression within the locus in N+2-expressing cells (Supplementary Figure S6A).

We conclude that a truncated form of CTCF composed of its N-terminal domain and two ZF-domains could be utilized for the directed modification of the chromatin loop profile.

### 2.4. N+2 Binding Partially Restores Chromatin Loops Lost After Deletion of the Endogenous CBS

**Figure 5:**
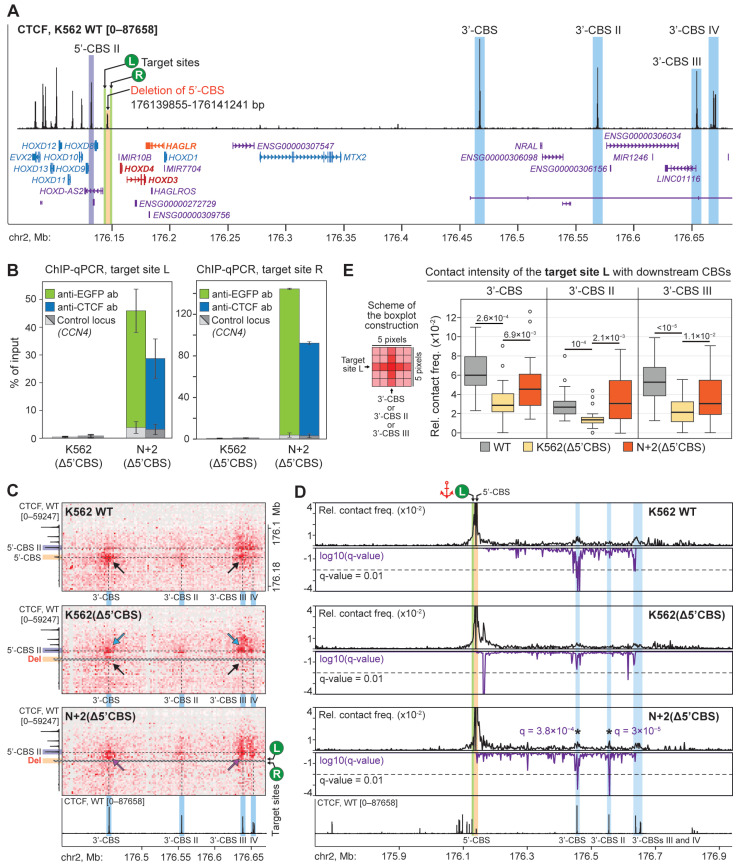
The recruitment of N+2 chimeric protein partially restores lost chromatin loops. (**A**) A scheme of the locus illustrating the positions of 5′-CBS deletion, surrounding CBSs, target sites for the N+2 recruitment in the K562(Δ5′CBS) cells (vertical green lines) and genes, differentially expressed between WT K562 and K562(Δ5′CBS) cells (protein-coding genes *HOXD4* and *HOXD3*, and gene of non-coding RNA *HAGLR* are highlighted in red and orange, respectively). (**B**) ChIP-qPCR analysis of the N+2 chimeric protein binding at the target sites near the 5′-CBS deletion. An amplicon within the *CCN4* locus was used as a negative control. The average value of two independent biological replicates and the standard error of the mean are shown. (**C**) Snapshots of the C-TALE heatmaps showing contacts of the 5′-CBS and 5′-CBS II with downstream CBSs. Position of the 5′-CBS deletion is shown with the gray dashed line. Black arrows indicate contacts of the 5′-CBS with 3′-CBS and 3′-CBS III in the K562 WT cells, and the loss of these contacts in the K562(Δ5′CBS) line. Blue arrows show the emergence of contacts of the 3′-CBS and 3′-CBS III with the 5′-CBS II after deletion of the 5′-CBS. Magenta arrows denote the increased contact frequency of the target site L with the downstream CBSs in the N+2-expressing K562(Δ5′CBS) cells. Data resolution—2 kb. (**D**) Virtual 4C profiles of the relative contact frequency of the target site L (marked with an anchor; top panels) and FDR-adjusted *p*-values (q-values) of the contacts (violet plots in the bottom panels). The significance threshold equal to 0.01 is indicated by a dashed line. Contacts of the target site L with 3′-CBS and 3′-CBS II in the N+2-expressing K562 (Δ5′CBS) cells are indicated with asterisks. (**E**) Boxplots showing distributions of the C-TALE counts in the areas of the heatmaps corresponding to contacts of the target site L with downstream 3′-CBSs (*n* = 25). Horizontal bold lines represent median values. *p*-values are calculated in the Mann-Whitney U test.

CBS deletions disrupt normal looping interactions in chromatin, resulting in the loss of enhancer-promoter contacts and violations of TAD boundaries in a number of pathologies [5]. To test the ability of the N+2 chimeric protein to restore a loop lost after CBS deletion, we established the K562(Δ5′CBS) cell line bearing a short (1386 bp) deletion, which removes 5′-CBS (Figure 5A). Deletion has been performed by the CRISPR-Cas9 system and confirmed by sequencing of the PCR product amplified with primers flanking the deleted fragment (Supplementary Figure S6D). K562(Δ5′CBS) cells exhibit no changes in size and morphology, and a minor decrease in division rate. Differential expression analysis revealed a significant upregulation of *HOXD3* (305-fold, *p* = 3.2 × 10^−6^), *HOXD4* (213-fold, *p* = 4.8 × 10^−9^) and *HAGLR* (24-fold, *p* = 7.1 × 10^−4^) genes (Supplementary Figure S6B). Interestingly, *HoxD3* and *HoxD4* are also upregulated in the proximal forelimb cells of the mouse embryo upon the deletion of the CBS, which is homologous to 5′-CBS [57]. In line with this observation, we also detected a decrease in H3K27me3 level in the K562(Δ5′CBS) cells within the 3′-half of the *HOXD* domain where these genes are located (Supplementary Figure S5B,C). Remarkably, in the 5′-part of the domain, H3K27me3 level is increased in the K562(Δ5′CBS) cells, and the boundary between these two areas coincides with the CBS in a forward orientation (Supplementary Figure S5C, marked with an asterisk).

Next, we designed two gRNAs (Supplementary Table S2) to recruit N+2 to both flanks of the deleted region. These gRNAs are separated by 871 bp in the K562(Δ5′CBS) genome and determine left (L) and right (R) target sites (Figure 5A). ChIP-qPCR with anti-EGFP antibodies (Figure 5B, green bars) and anti-CTCF antibodies (Figure 5B, blue bars) confirmed effective and selective recruitment of the N+2 to both targets, with a relatively higher level of the protein binding at the target site R.

In the wild-type K562 cells, 5′-CBS established strong contacts with two downstream CBSs, which are referred to as 3′-CBS (mentioned above) and 3′-CBS III (Figure 5C, upper line). Contacts with two other CBSs (3′-CBS II and 3′-CBS IV) are detectable, but relatively weak. Deletion of the 5′-CBS results in a complete loss of interactions with 3′-CBS and 3′-CBS III (Figure 5C, middle line). This is supported by the analysis of virtual 4C profiles constructed for the viewpoint adjacent to the 5′-CBS and retained after its deletion (contains the target site L, genome coordinates chr2:176,136,800–176,138,800 bp, Figure 5D). In the K562 (Δ5′CBS) line, contrary to the wild-type cells, 3′-CBS and 3′-CBS III establish bright contacts with 5′-CBS II, which is located upstream of the 5′-CBS (Figure 5C, middle line). We found that the N+2 recruitment partially reverses these changes induced by the 5′-CBS deletion. The C-TALE heatmap shows an increase in contact frequency of the target site L with 3′-CBS and 3′-CBS III in the N+2-expressing K562(Δ5′CBS) cells (Figure 5C, bottom line). For the contact with the nearest 3′-CBS, this is also manifested in a statistically significant peak (q = 3.8 × 10^−4^) on the virtual 4C profile (Figure 5D, bottom line). Interestingly, in the N+2-expressing K562 (Δ5′CBS) cells, the 4C profile demonstrated an increase in contact frequency between the target site L and 3′-CBS II (Figure 5D; q = 3 × 10^−5^). We have also supported the results of visual inspection of the C-TALE heatmaps by a quantitative assessment of the contact frequency of the target site L with 3′-CBS, 3′-CBS II and 3′-CBS III. To this end, we calculated the number of contacts within areas of 5 × 5 pixels centered at the corresponding interactions (Figure 5E). This analysis revealed that the N+2 recruitment results in an increase of contact frequency between the target site L and downstream CBSs, confirming that the N+2 chimeric protein is able to mediate distant interactions in chromatin. Finally, we did not detect changes in the H3K27me3 profile and transcription level of *HOXD* genes in N+2-expressing K562(Δ5′CBS) line as compared to control K562(Δ5′CBS) cells (Supplementary Figures S5B and S6C). Thus, a partial restoration of the lost loops did not reverse changes in the chromatin epigenetic state and transcription of the affected genes.

## 3. Discussion

Targeted modulation of the chromatin spatial structure is a relevant task of 3D genomics since distant interactions between remote genome elements represent an important layer of transcription regulation. A vast majority of the techniques developed to date are based on the usage of chimeric proteins consisting of dimerization domains and DNA-binding modules such as arrays of zinc-finger domains and dCas9 [20,21,25,58,59]. Recruitment of such chimeras to target loci allows formation of either permanent chromatin loops in the case of constitutive dimerization domains (such as LDB1 dimerization domain [60]), or transiently-formed interactions whose formation could be controlled by small dimerization-inducing molecules [59] or even by an exposure of cells to blue light [58]. An apparent disadvantage of such technologies is their “artificiality” manifested in the necessity of using inductors of loop formation. In this regard, utilization of a natural architectural protein CTCF looks like an attractive alternative. CTCF is a part of the natural communication system in chromatin consisting of a cohesin “motor” that carries out extrusion and a “stop signal” represented by CTCF-binding sites frequently located in the vicinity of genome regulatory elements. Previously published observations suggest that the recruitment of a full-length CTCF to chromatin via fusion with DNA-binding modules results in loop formation between the target site and nearby loci [20,21]. However, our data suggest that the usage of a full-length CTCF for the construction of chimeric proteins possesses considerable side effects manifested in cytotoxicity, at least in K562 cells (Supplementary Figure S1D). This is in line with the reported cytotoxicity of CTCF overexpression for these cells [48]. ChIP-seq profiling revealed that the chimeric protein consisting of a full-length CTCF retains the ability to bind endogenous CBSs (Figure 2D). This could induce transcription dysregulation throughout the genome similar to that upon CTCF overexpression [61], and as a consequence, result in lower cell viability. Interestingly, the viability of the cells expressing the N+7 and N+11 chimeric proteins was comparable to that of the control and N+2-expressing cells. Since both N+7 and N+11 proteins contain all ZF domains required for the core motif binding, some other factors besides the increase of CTCF abundance at CBSs could contribute to the toxicity of the Full chimeric protein. One possible reason is that the Full chimeric protein contains the CTCF C-terminal domain, which is responsible for the recruitment of RNA polymerase II to CBSs [62]. It is relevant to assume that the Full chimeric protein expression would recapitulate CTCF overexpression and potentially influence transcription profile genome-wide by an excessive RNA polymerase recruitment to chromatin. Our data suggest that the N+2 chimeric protein could be a more appropriate molecular instrument for the 3D genome modification, because it does not induce cytotoxicity and selectively binds to the target site, but not to endogenous CBSs. While distant contacts mediated by the N+2 recruitment are relatively weak (which potentially explains why the N+2 recruitment to the vicinity of the deleted CBS did not reverse changes in epigenetic and transcription profiles), their formation relies on the naturally operating mechanism of cohesin-driven extrusion and its termination at CTCF barriers. This is confirmed by the fact that the target site for the N+2 chimeric protein accumulates cohesin (Figure 4). Notably, a previous study used the CTCF N-terminal domain with two proximal ZFs fused to artificial DNA-binding ZFs, which did not detect cohesin enrichment at the binding sites of this protein [25]. Relatively weak contacts also suggest that the majority of extrudes do not terminate at the N+2-occupied target site. We suppose that, among other factors, the absence of the ZF10 in the N+2 chimeric protein contributes to a low efficiency of the cohesin stalling. ZF10 is responsible for the RNA binding, and this is critical for loop formation at least in some genome loci [27]. Another potential reason for the relatively low efficiency of the N+2-mediated loop formation is that this protein lacks a number of sites for PTMs, which are located in the C-terminal part of the full-length CTCF [63]. While the role of these PTMs in the cohesin stalling is not described to date, it should be taken into account.

## 4. Materials and Methods

### 4.1. Cell Cultures

The K562 cell line was maintained in a high-glucose DMEM medium (PanEco, Moscow, Russia) supplemented with 10% of fetal bovine serum (FBS), 1000 units/mL penicillin and 1000 units/mL streptomycin. K562(Δ5′CBS) cell line with deletion of CTCF binding site in *HOXD* locus was obtained by electroporation of plasmids encoding the Cas9 protein and two gRNAs (Supplementary Table S1). The gRNAs were selected by the CRISPOR program (https://crispor.gi.ucsc.edu, accessed on 3 April 2023) [64], cloned into the phU6 plasmid (Addgene #53188, Watertown, MA, USA) and tested by the ENIT method [65] with the previously selected gRNA to the *MYC* gene. After 2 days, single cells were sorted into five 96-well plates, and genomic DNA was checked for the deletion via PCR with pairs of primers placed both inside and outside the deleted fragment (see HOXD-indel and HOXD-del pairs in Supplementary Table S3).

### 4.2. Electroporation

Electroporation was performed using the NEON system (Thermo Scientific, Waltham, MA, USA). For each electroporation, 1.5 million cells were washed twice with DMEM medium (without FBS and antibiotics), once with 1× PBS solution, and resuspended in 105 μL of R buffer (Thermo Scientific, Waltham, MA, USA) with 12 μg of plasmid DNA (8 μg for Cas9 plasmid and 2 μg for each gRNA plasmid). A total of 4 pulses (10 ms each at 1350 V) were performed. After electroporation, cells were placed in 3 mL of DMEM medium supplemented with 20% FBS (without antibiotics).

### 4.3. Constructing Plasmids Encoding Parts of the CTCF Protein Fused with dCas9 and EGFP

All plasmids were created by modification of the pSLQ1658-dCas9-EGFP (Addgene #51023) plasmid, encoding dCas9 fused with EGFP.

Fragments of cDNA encoding parts of *CTCF* CDS were obtained by extracting total RNA of 1 mln of K562 cells, reverse transcription and PCR with specific primers (Supplementary Table S3). *E.coli STBL3* cells were transformed with the plasmids and were cultivated at 32 °C overnight.

### 4.4. Preparation of Cells for the Experiments

For each experiment, electroporations of 9 aliquots of K562 or K562(Δ5′CBS) cells were performed, 1.5 million cells per aliquot. A total of 2 days after electroporation, 2 million cells were collected for the C-TALE, 4 million for the ChIP-qPCR and ChIP-seq, 2 million for the RNA extraction and 1 million for the Western Blotting.

For the Western Blotting, cells were washed once with empty DMEM, twice with 1× PBS and lysed in 1 mL of Western RIPA buffer (150 mM NaCl, 1% Triton X-100, 0.5% Na-deoxycholate, 0.1% SDS, 50 mM Tris-HCl, 1 mM PMSF, 1× PIC) for 10 min.

For the C-TALE and ChIP-seq experiments, cells were washed once with empty DMEM, resuspended in 1 mL of empty DMEM and fixed with formaldehyde for 10 min (2% for the C-TALE, 1% for the ChIP-seq). The reaction was quenched by adding 70 μL of 2M Glycine. Cells were pelleted, washed twice with 1× cold PBS and frozen at −150 °C.

For the RNA-seq, cells were washed once with empty DMEM and 1× cold PBS, pelleted and resuspended in 1 mL of TRIzol reagent, incubated for 5 min and frozen at −80 °C.

### 4.5. Western Blot

Lysed cells were sonicated using VirSonic 100 (VirTis) for 30 s at power 10 via. Protein amount was measured by absorption at 595 nm with Breadford solution. Each sample was mixed with 4× SLB (with β-mercaptoethanol and bromophenol blue), incubated at 100 °C for 10 min, aliquoted and frozen at −80 °C. Protein samples were resolved on a gradient 4%-6%-8% SDS-polyacrylamide gel electrophoresis. Gel was incubated in the transfer buffer (25 mM Tris-HCl, 192 mM Glycine, 20% Methanol) for 10 min. Protein samples were transferred from the gel to the Hybond-P membrane, and incubated overnight with anti-CTCF antibodies (Active Motif, Carlsbad, CA, USA, cat. 61311) at a 1:2000 dilution at 4 °C. The Membrane was washed thrice with PBST, incubated with the secondary antibodies (Rabbit; Cytiva, Marlborough MA, USA, cat. NA934-100UL) at a 1:10,000 dilution for 1 h. Membrane was washed 5 times in PBST and visualized by SuperSignal West Dura Extended Duration Substrate (Thermo Scientific, Waltham, MA, USA).

### 4.6. C-TALE Library Preparation 

The procedure was performed as previously described [51]. Briefly, pellets of fixed cells were quickly defrozen and lysed in isotonic buffer (50 mM Tris-HCl pH 8, 140mM NaCl, 1% Triton X-100, 0.5% NP-40, 1× Protease Inhibitor Cocktail (Bimake, Gravesano, Switzerland)) for 15 min on ice, washed twice with 200 μL of 1.1× buffer for the DpnII restriction enzyme (New England Biolabs, Ipswich, MA, USA). A total of 3.04 μL of 20% SDS was added to each sample with subsequent incubation for 1 h at 1400 rpm at 37 °C. After incubation, 52.7 μL of Triton X-100 was added to each sample, together with 330 μL of 1.1× buffer for DpnII restriction enzyme (New England Biolabs, Ipswich, MA, USA, 50,000 U/mL), followed by one more incubation with constant shaking (1400 rpm) for 1 h at 37 °C. After that, 10 μL of DpnII restriction enzyme was added to each sample, followed by overnight incubation at 37 °C.

Samples were washed thrice with 200 μL of 1× T4 DNA ligase buffer (New England Biolabs, Ipswich, MA, USA) and resuspended in 300 μL of 1× T4 DNA ligase buffer. A total of 15 μL of T4 DNA ligase (Thermo Scientific, Waltham, MA, USA, 5U/μL) was added to each sample, followed by incubation for 8 h at 16 °C with constant shaking. After incubation, 15 μL of Proteinase K solution (20 μg/μL) was added to each sample, together with 17.37 μL of 20% SDS. Samples were incubated overnight at 65 °C.

DNA was extracted with phenol:chloroform mixture (1:1) and ethanol-precipitated with the use of 100 μg of tRNA (Sigma-Aldrich, Burlington, VT, USA) and 100 μg of glycogen (Thermo Scientific, Waltham, MA, USA, 20 mg/mL) as co-precipitators. DNA was dissolved in 10 mM Tris-HCl and treated with 50 μg of RNAse A (Thermo Scientific, Waltham, MA, USA, 10 mg/mL) at 37 °C for 45 min.

Samples were sonicated for 4 times (power 15; 30 sec each pulse + 2 min pause) via VirSonic 100, concentrated in Amicon 30K filters (Sigma-Aldrich, Burlington, VT, USA) and incubated for 30 min with 5 μL of PNK (New England Biolabs, Ipswich, MA, USA, 10,000 U/mL), 4 μL of T4 DNA polymerase (New England Biolabs, Ipswich, MA, USA, 3000 U/mL) and 1 μL of Klenow fragment (New England Biolabs, Ipswich, MA, USA, 5000 U/mL) in 1× T4 DNA ligase buffer with addition of dNTPs to repair DNA ends. Samples were purified on MagPure A4 XP magnetic beads and proceeded to incubation at 37 °C for 30 min with 5 μL of Klenow fragment without 3′-exonuclease activity (New England Biolabs, Ipswich, MA, USA, 5000 U/mL) and 5 μL of 10 mM dATPs (Thermo Scientific, Waltham, MA, USA) in 1× NEB2 buffer (New England Biolabs, Ipswich, MA, USA) to add dATP to ends of the DNA fragments. Samples were once again purified on MagPure A4 XP magnetic beads and mixed with 0.5 μL of TruSeq Illumina Adapters (Illumina, San Diego, CA, USA) and 2 μL of T4 DNA ligase in 1× T4 DNA ligase buffer to ligate Illumina adapters. Samples were incubated overnight and then amplified with KAPA Taq ReadyMix (KAPA Biosystems, Merck, Darmstadt, Germany) and Illumina adapter primers (see Illumina-for primer and Illumina-rev primer in Primer Table). After amplification, samples were hybridized with the biotin-labeled baits obtained from BACs covering the *HOXD* locus for 40 h (Supplementary Figure S2). After that, products of hybridization were captured by Streptavidin-coated magnetic beads Dynabeads MyOne C1 (Invitrogen, Thermo Scientific, Waltham, MA, USA), washed of the non-specifically bound DNA, PCR-amplified, and hybridization was repeated. After the 2nd hybridization, PCR-amplified DNA was sequenced on the NovaSeq 6000 (Illumina, San Diego, CA, USA) by 100-nt paired reads.

### 4.7. C-TALE Data Processing and Analysis

Quality control of the reads was performed using FastQC (Version 0.11.8). The raw reads were mapped to the 175,634,800–176,937,632 bp DNA sequence from the human hg38 assembly using *hiclib* [66]. The same tool was used to filter out reads that mapped in close proximity to the DpnII restriction sites (5 bp), reads from the same restriction fragment, and possible PCR duplicates. Resulted contact maps were iteratively corrected using *cooler* (v0.8.11) (https://github.com/mirnylab/cooler, accessed on 10 July 2023). The 4C profiles were generated from cool-files using custom code (code available upon request), and q-values for interactions were calculated using the LASCA pipeline [67].

### 4.8. ChIP-qPCR and ChIP-Seq Library Preparation

Cells were quickly defrozen and lysed in the RIPA buffer for 15 min on ice. Chromatin was sonicated to 100–500 bp, concentrated in Amicon 30K filters (Sigma-Aldrich, Burlington, VT, USA) and diluted with RIPA buffer to 500 μL; 10% was taken as an input control. Chromatin was incubated with antibodies against (EGFP (Evrogen cat. AB011, Moscow, Russia), SMC3 (Abcam cat. ab9263, Waltham, MA, USA), CTCF (ActiveMotif cat. 61311), IgG (Jackson ImmunoResearch cat. 011-000-002, West Grove, PA, USA)) overnight at 4 °C on a rotor. The next day, chromatin was incubated with pre-washed A/G ChIP beads (Thermo Scientific, Waltham, MA, USA) for 6 h at 4 °C on a rotor. After incubation, samples on the beads were washed and resuspended in 100 μL of PBS. To each sample, 4 μL of 20% SDS and 100 μg of Proteinase K were added. All samples and controls were incubated at 65 °C overnight with constant shaking. DNA was extracted with phenol:chloroform solution (1:1) and ethanol-precipitated with tRNA and glycogen as co-precipitators. DNA was dissolved in 100 μL of 10 mM Tris-HCl and treated with 50 μg of RNAse A at 37 °C for 45 min.

qPCR with primers and TaqMan probes to the target site and control locus was performed with DreamTaq DNA polymerase (Thermo Scientific, Waltham, MA, USA, 5 U/μL).

ChIP-seq libraries were prepared in the same way as C-TALE libraries with DNA end repair, A-tailing, Illumina adapter ligation, amplification and a single hybridization step.

### 4.9. ChIP-Seq Data Processing 

ChIP-seq raw reads were mapped (hg38 human genome assembly) using *bwa* (version 0.7.18-r1243-dirty) [68] sorted and deduplicated using *samtools* (version 1.21) [69] (http://www.htslib.org, accessed on 1 February 2024). Deduplicated files were transformed to BigWig format using *deepTools* (version 3.5.5) [70] (https://deeptools.readthedocs.io, accessed on 1 February 2024) and visualized in the UCSC Genome Browser [54].

### 4.10. RNA-Seq Library Preparation

Samples were defrozen quickly. A total of 200 μL of chloroform was added to each sample, mixed intensively and incubated for 3 min. Samples were centrifuged at 20,000× *g* for 15 min (4 °C). An equal amount of isopropanol was added to the aqueous phase, and the samples were incubated for 10 min at room temperature and centrifuged at 20,000× *g* for 10 min (4 °C). The supernatants were removed and the residues were washed with 70% EtOH (prepared with DEPC-mQ), air-dried for 10 min and resuspended in 175 μL of RNAse-free water. A total of 20 μL of 10× DNAse I buffer (Roche, Indianapolis, IN, USA), 5 μL of DNAse I (Roche, 10 U/μL) and 5 μL of RiboLock RI (Thermo Scientific, Waltham, MA, USA, 40 U/μL) were added, and samples were incubated for 1 h at 37 °C. RNA was extracted with an equal volume of acidic phenol:chloroform (pH 4.3) solution. RNA was precipitated by adding 0.7 volume of isopropanol, pelleted at 20,000× *g* for 15 min (4 °C) and then resuspended in 35 μL of RNAse-free water, aliquoted and stored at −80 °C. A total of 1 μg of RNA was used for the library preparation by NEB Next Ultra II Directional RNA Library Prep Kit for Illumina (New England Biolabs, Ipswich, MA, USA). After the cDNA ends preparation step, each sample was mixed with 4.5 μL of RNAse-free water, 7.5 μL of 10× T4 DNA ligase buffer, 2 μL of T4 DNA ligase and 1 μL of TruSeq Illumina adapter. Samples were incubated at room temperature overnight to ligate adapters. The next day, purification on MagPure A4 XP magnetic beads (Magen Biotechnology Co., Ltd., Guangzhou, China), PCR amplification and hybridization steps were performed similarly to the C-TALE libraries with a single hybridization step.

### 4.11. RNA-Seq Data Processing and Analysis

Raw reads were processed using the STAR aligner (v.2.7.11b) [71], *samtools* (version 1.21) [69] and *featureCounts* (v2.0.1) [72] (https://subread.sourceforge.net, accessed on 1 February 2024). Counted reads were processed and normalized in DEseq2 (v.1.42.1) [73] using RStudio (2023.12.1 build 402) working environment with a custom R script (R version 4.3.2).

## Data Availability

Raw and processed C-TALE, ChIP-seq and RNA-seq data are available in the GEO repository under the accession number GSE300308.

## Supplementary Materials

The following supporting information can be downloaded at: https://www.mdpi.com/article/10.3390/ijms26157446/s1.
