# Supplementary material for: Ectopic Recruitment of the CTCF N-Terminal Domain with Two Proximal Zinc-Finger Domains as a Tool for 3D Genome Engineering
Source: Int J Mol Sci. 2025 Aug 1;26(15):7446. doi: 10.3390/ijms26157446 (PMC12347799; doi:10.3390/ijms26157446)
Supplement: Supplementary file 1 [file ijms-26-07446-s001.zip › ijms-3744216-Supplementary Figures.pdf]

SUPPLEMENTARY FIGURES

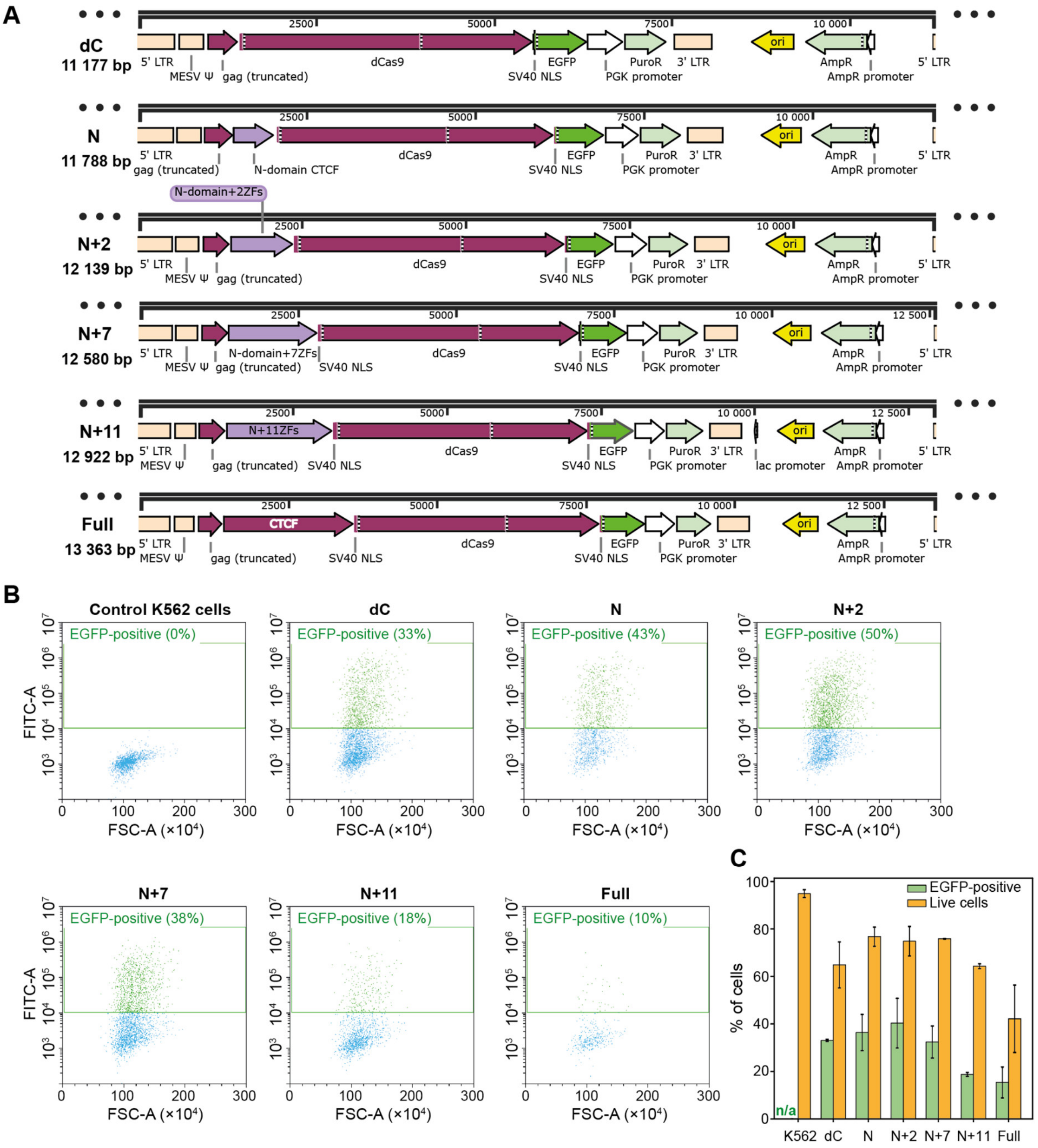

Supplementary Figure S1 (related to Figure 1). Expression constructs for the chimeric proteins and analysis of protein expression and cell viability. (**A**) Schematic representation of the expression plasmids, encoding chimeric proteins. (**B**) Flow cytometry analysis of the control and chimeric protein-expressing K562 cells showing the proportion of EGFP-positive (FITC-A) and live (FSC-A) cells. (**C**) Proportion of EGFP-positive and live cells. The average value of two independent biological replicates and standard error of the mean are shown.

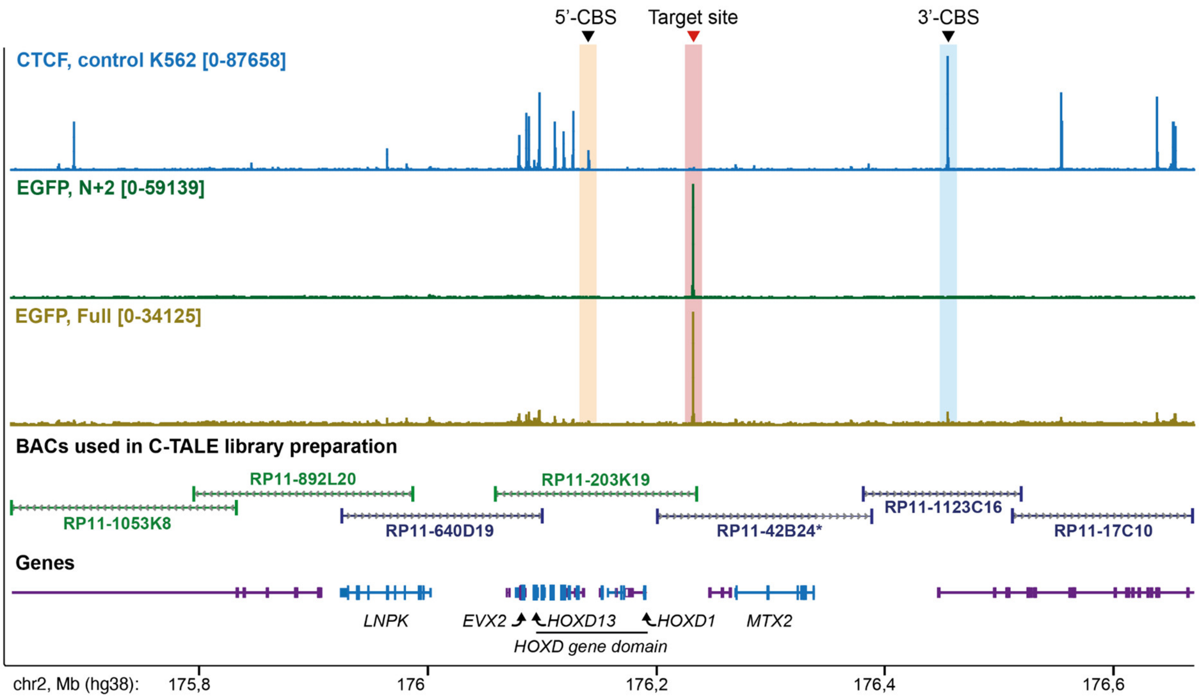

Supplementary Figure S2 (related to Figure 2). ChIP-seq profiling of the N+2 and Full chimeric proteins within the analyzed genome region (chr2:175,634,800 bp - 176,670,160 bp). ChIP-seq analysis of N+2 and Full chimeric protein binding at the target site. Upper line: control K562 cells, anti-CTCF antibodies were used; middle and bottom lines: N+2- and Full-expressing cells, respectively; anti-EGFP antibodies were used. Bacterial artificial chromosomes (BACs) used for the C-TALE library preparation and for the enrichment of ChIP-seq and RNA-seq libraries (RP11-42B24, labeled with an asterisk , is present in the hg19 genome assembly and absent in the hg38 assembly). Protein-coding and non-coding RNA genes are highlighted in blue and violet, respectively.

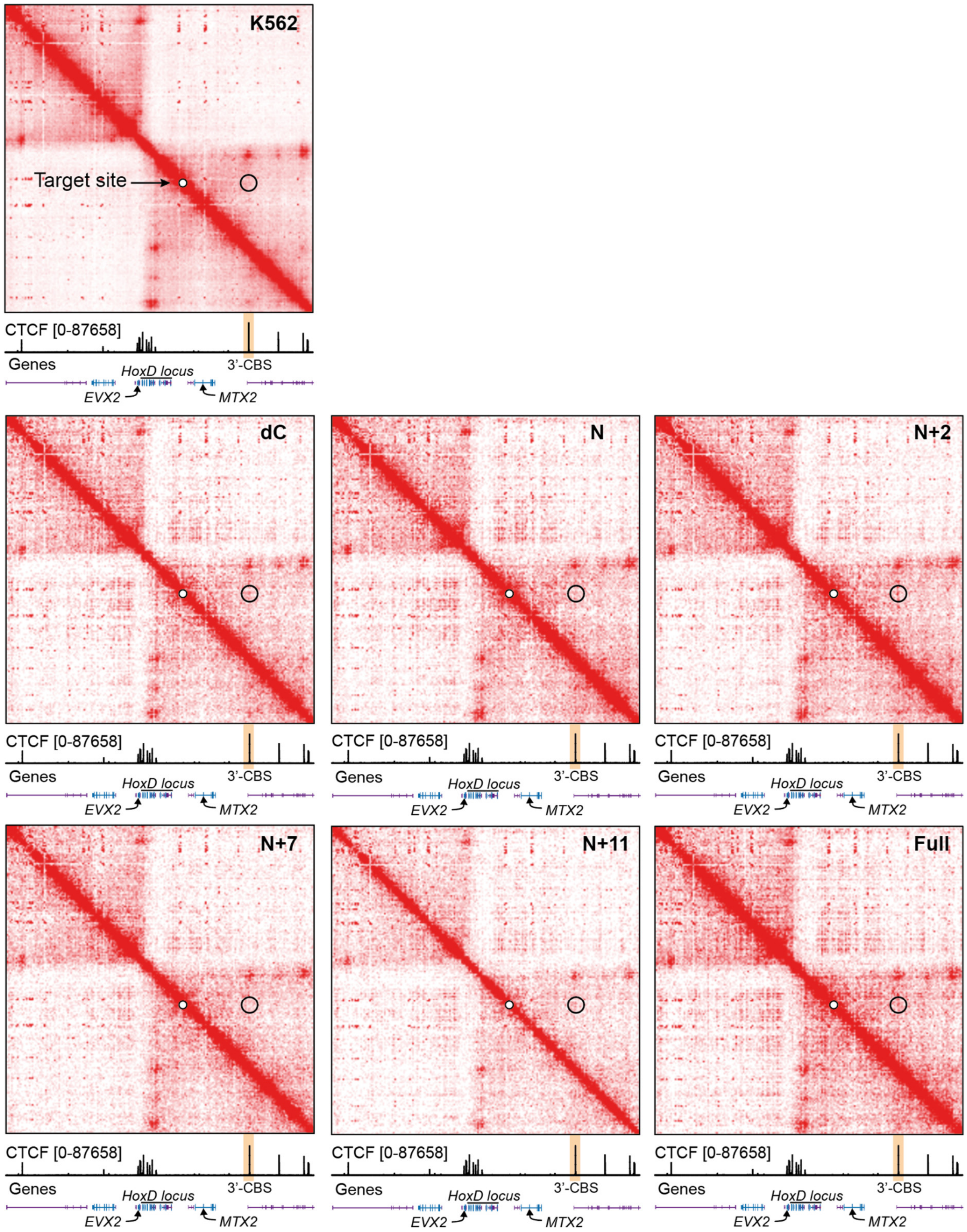

Supplementary Figure S3 (related to Figure 3). The whole-locus C-TALE heatmaps. White circle at the map diagonal shows the target site for the recruitment of the chimeric proteins.

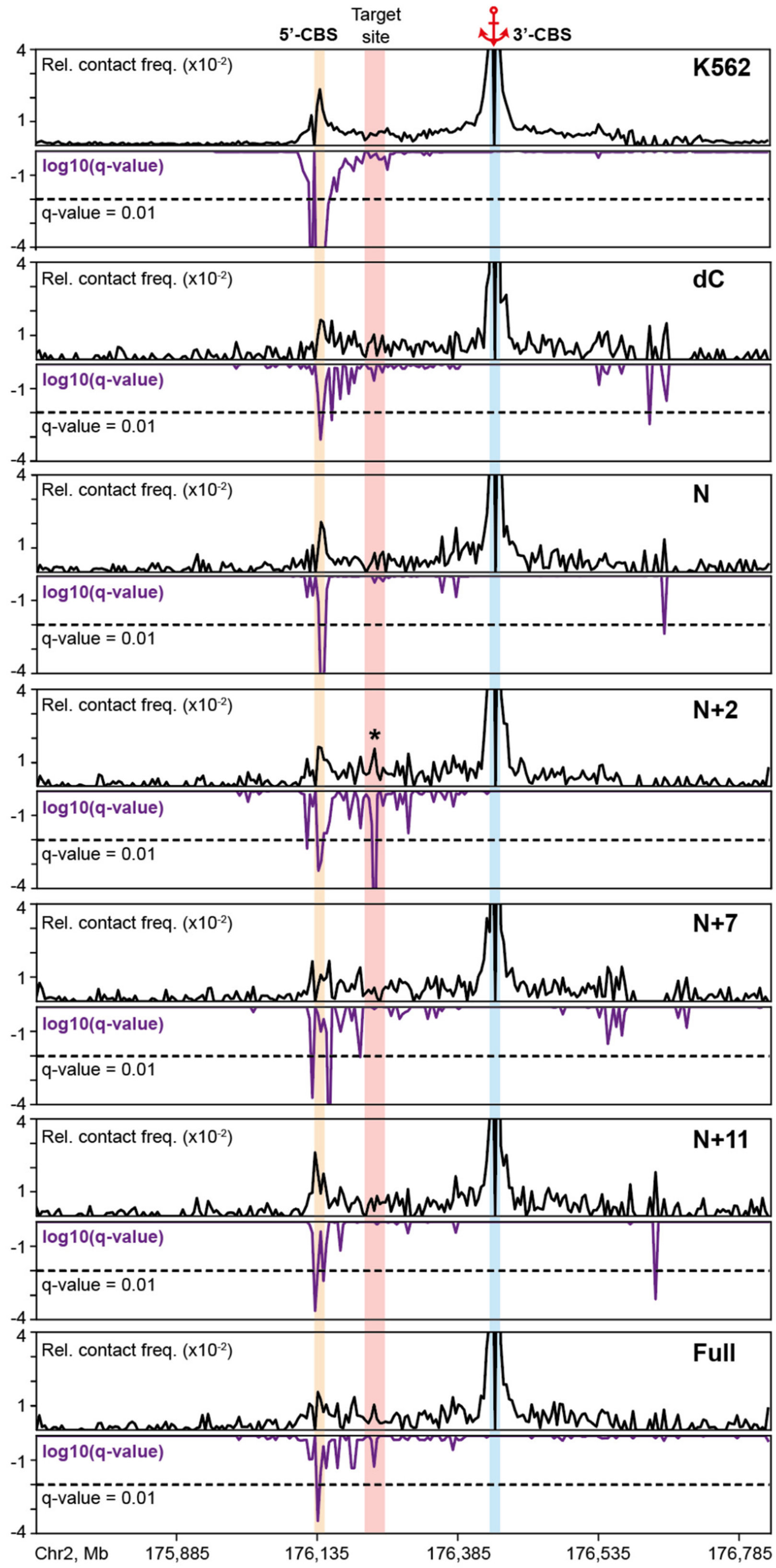

Supplementary Figure S4 (related to Figure 3). Virtual 4C-profiles of the relative contact frequency of the 3'-CBS (upper panels) and FDR-adjusted p-values (q-values) of the contacts (violet plots in the bottom panels). A contact between the 3'-CBS and the target site in the N+2-expressing cells is indicated with an asterisk.

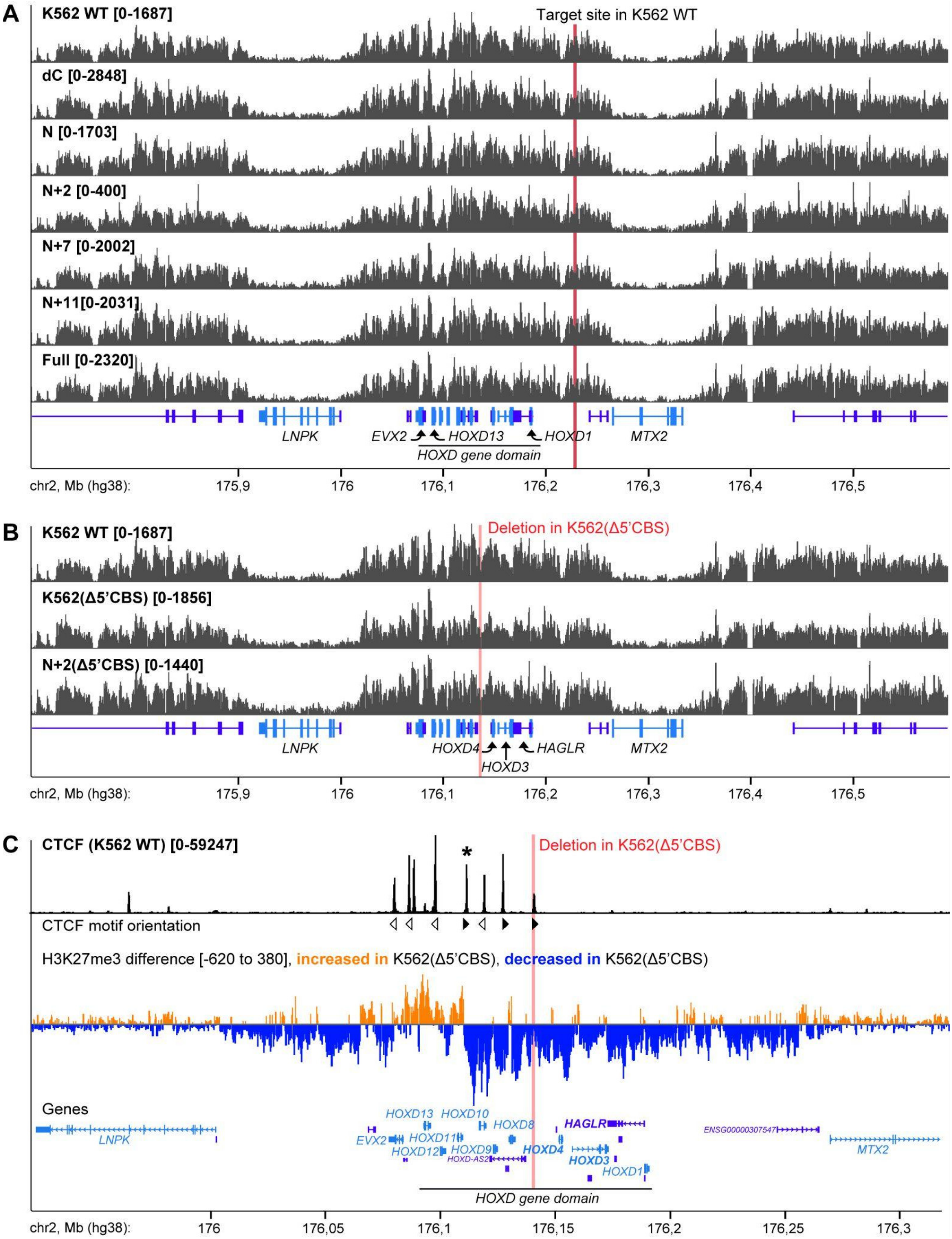

Supplementary Figure S5 (related to Figures 3 and 5). H3K27me3 ChIP-seq profiles across the *HOXD* locus in the wild-type K562 cells (K562 WT, **A**) and in K562 cells with deleted 5'-CBS (K562(Δ5'CBS)), **B**). (**C**) Difference in H3K27me3 levels between the K562(Δ5'CBS) and K562 WT cells. CTCF ChIP-seq profile and CTCF motifs show that the boundary between parts of the *HOXD* gene domain with opposite changes in H3K27me3 abundance coincides with the CBS in a forward orientation (asterisk).

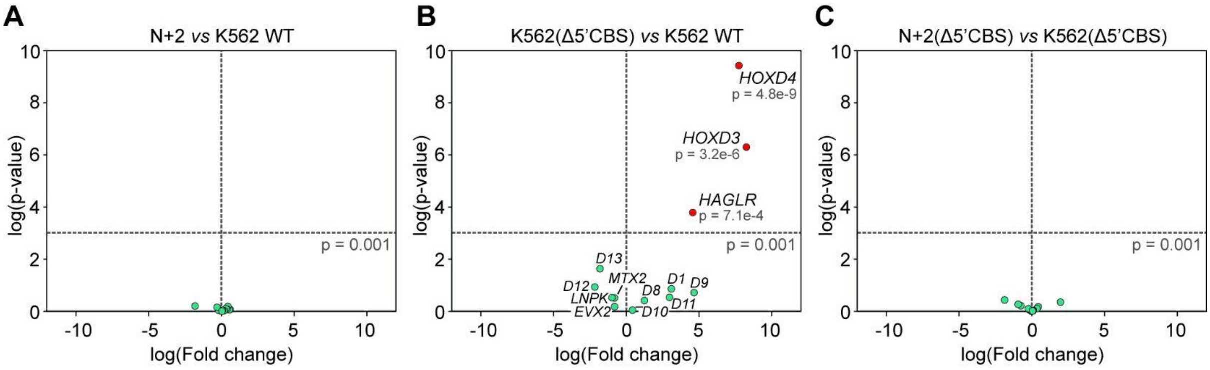

D

Genomic sequence	Genomic coordinates (hg38, chr2)	
gcaaatcaga aaggacagtt gaagctttta atacatgttt tcctgaaaaa	176139668	
gaaaagtgtg tttcctgctt cttgagatga ggttcccaag ctcctctgca	176139718	
TCCAGGCGTA GAAGCAGTGC GCGGTACCTT ATCCGCACTG CGCGGTGCAA	176139768	
GCCGGCCGCA CAGCTCACGG TCCCGCAGCG CCCGCCACAC ACCCGCGCCA	176139818	PCR-product
GAGGTCCAGC GCATGTGCAG TGAAATGGCC TAGCCCggga attcgatcgg	176139868	
caaagcacgg gtgtgggggc gggggggtgg gggggggcga gtggactcga	176139918	
gcaggaagat acacccgatc tttccattct ccctccgtca aggctcgcta	176139968	
ctcatcttgc ctttatctca gccttccctg tatagctgaa cagttctacc	176140018	
tttggctaca tttccaatca ttatgactta tcacctcacc ccagtttaaa	176140068	
tctgtacgct agacatagc tgaaaatacg agacgagggc caaaggaaac	176140118	
actcttcttt ccagtttata tatatatata gcatatattt gcccctcggg	176140168	
gaaggctgta gatttttttt tttcagcctg ctgctactac tttgggaaaa	176140218	
ggccttttct cctggcgcct gttcaaaccg ccacacttca ggccaatcct	176140268	
tcggcctgac cattcccgga tcttgttatg ccagcacagc acattcgagg	176140318	
gctctggggg gcagcagctc ggcgaagagc agtcaaataa ataaataaat	176140368	
aaacaaataa atgaacggac atataagggc ttttcccgcc tgggttcgct	176140418	
taaataatgg gggacaagcg cctctgggcc acgagcttgt tgtttgtcct	176140468	
gggatggggg gtaggggatg agaggtggcc tcaggtccct cttgctcctt	176140518	
ccagccgccc caggggacac tgtagaacca gcctctggtg cggggagacc	176140568	
tccgcctgcc ggaggaagtc ccagcctcag gtttccgggg ctggtggtct	176140618	
ccctgctctg ctgtctggcg cctcgtccag gaggaggcgc tgtaggacaa	176140668	CTCF motif
gcgtctcgcc ggctgcggcg ggccggggag ggcgggagcg gagggatcgg	176140718	
tacccgctcc tcccttcccg cgcactctac ctccgcgcgc caagtgtgtg	176140768	
gggcctcgaa caagggtcga cggtgctgcg ccccgtcccc caaggggttc	176140818	
ctttccccag actggcagtg gaagcagctc cgaggcctgg gagcctaggg	176140868	
ccgttccggg ttggcgggcc acctcgcttt ctccgcgctc ccggaggggg	176140918	
cgcgatttcc tcgtgctgcg cttgtaggag cagcaacttg aatcctgtgt	176140968	
ggggcaagct gggcccaatc cgcggatccc aaccagcccc tcccgctggg	176141018	
ccgtgggtct cgtcatttac cttgcccagg attcctggta cccagaatgg	176141068	
acagccttga aaagcacatc ctttagtgcc tcctccaaca ggctgatggg	176141118	
ccttgccctt gctcacttag aacaggacct gggtcctggg actccctgga	176141168	
gactcagagc ttgggtaaat cccatttgct ctgcagctca agagaactag	176141218	
ccagatgcgc aTGTCTACTC CACACCACCC AACCACTCTT CTATCACCAC	176141268	
TTCGCTCATT TCAGGCCTTT TGTGGGCTCC ACTGGGCACT CCTCGCACCA	176141318	PCR-product
GGTTCAAGAG CCAGCGACAC CTTacttaaa taatgctctg acccaatccc	176141368	
actgatcctc cgttatctct tggcaatgca gtcttttgtc cccctctccc	176141418	
caccgccttc tctagggtaa tga		

Supplementary Figure S6 (related to Figures 3, and 5). Differential gene expression analysis. (**A-C**) Vulcano plots for the wild-type N+2-expressing K562 cells (**A**), K562 cells with deleted 5'-CBS, K562(Δ5'CBS) (**B**) and for the N+2-expressing K562(Δ5'CBS) cells (**C**). Genes with p-value < 0.001 and two-fold change in expression level are considered as differentially expressed genes (highlighted in red). (**D**) Deletion of 5'-CBS. The CTCF-binding motif is highlighted in red. Amplified PCR-product is highlighted in blue. The deleted region is located between the two parts of the PCR product.
